# Proteus Mediastinitis Causing Fatal Pseudoaneurysm following Aortic Valve Replacement

**DOI:** 10.1155/2013/920327

**Published:** 2013-11-28

**Authors:** Sarfraz Nazir, John Jeffery, Alexandra-Alice Tenovici, Horace D'Costa

**Affiliations:** ^1^Department of Radiology, Horton General Hospital, Oxford University Hospitals NHS Trust, Oxford Road, Banbury, Oxfordshire OX16 9AL, UK; ^2^Department of Surgery, Horton General Hospital, Oxford University Hospitals NHS Trust, Oxford Road, Banbury, Oxfordshire OX16 9AL, UK

## Abstract

We report an unusual case of Gram-negative mediastinitis following aortic valve replacement via median sternotomy. The patient presented two months after surgery following a urinary tract infection in septic shock with a discharging sternal wound and blood cultures positive for *Proteus mirabilis*. Imaging revealed a large anterior mediastinal abscess and aortic pseudoaneurysm which subsequently ruptured resulting in fatality. Gram-negative mediastinitis is a rare complication of cardiac surgery that can present late following initial clinical improvement and should be considered when “remote site” infections are present. Computerised Tomography scanning has a role to play in the identification of this.

## 1. Introduction

Mediastinitis is a recognised complication following median sternotomy. Despite relatively low reported incidences of approximately 1–4%, postoperative mediastinitis poses a detrimental effect on morbidity and survival, with mortality rates as high as 40% [1]. A plethora of studies exist attributing a whole host of risk factors in the pathogenesis of this condition, sometimes with conflicting views. It is proposed that development of mediastinitis is a multifactorial process involving both host and perioperative factors [2]. Most cases of mediastinal wound infections are attributed to gram-positive pathogens with *Staphylococcus aureus* and *Staphylococcus epidermidis* isolated in 70 to 80% of the cases [3].

Another complication of cardiac surgery is pseudoaneurysm formation involving the ascending aorta. This is rare with a reported incidence of less than 0.5% [4, 5]. These false aneurysms represent a dreaded complication, with rupture as the most deleterious consequence. Postoperative development of aortic pseudoaneurysms is commonly encountered at sites of aortic wall disruption with aortotomy sites being the most commonly implicated following aortic valve replacement.

We report a case of an occult mediastinal abscess presenting in association with a ruptured mycotic pseudoaneurysm of the ascending aorta in the setting of perioperative infection due to a gram-negative pathogen.

## 2. Case Report

An 84-year-old female with a known history of congestive cardiac failure, hypertension, and stable angina was initially seen in the Emergency Department at Horton General Hospital, Banbury. She presented with an acute onset of rapidly worsening exertional dyspnoea and reduced exercise tolerance. She was subsequently diagnosed with critical aortic valve stenosis following transthoracic echocardiography and underwent an urgent aortic valve replacement. The immediate postoperative course was uneventful except for the development of a pericardial effusion requiring drainage on the 10th day. Microbiological analysis of the pericardial fluid yielded cultures of the gram-negative pathogen, *Proteus mirabillis*. The patient completed a full course of intravenous gentamicin for 3 weeks with serial negative blood cultures. The wound site appeared to be healing well throughout this time. Further transesophageal echocardiography revealed no significant pericardial effusion and the patient subsequently made a good recovery and was discharged. Further cross-sectional imaging was not performed at this time.

Four weeks following discharge from hospital (two months postoperatively), the patient represented with fever, worsening dyspnoea at rest, and light headedness. She was clinically in septic shock and physical examination revealed an erythematous, warm anterior chest wall alongside a fluctuant swelling overlying the sternotomy scar with some purulent discharge. Laboratory data included a severe normocytic anaemia, neutrophilic leukocytosis, and significantly raised inflammatory markers. Subsequent blood cultures grew meropenem sensitive *Proteus mirabillis*.

**Figure 1:**
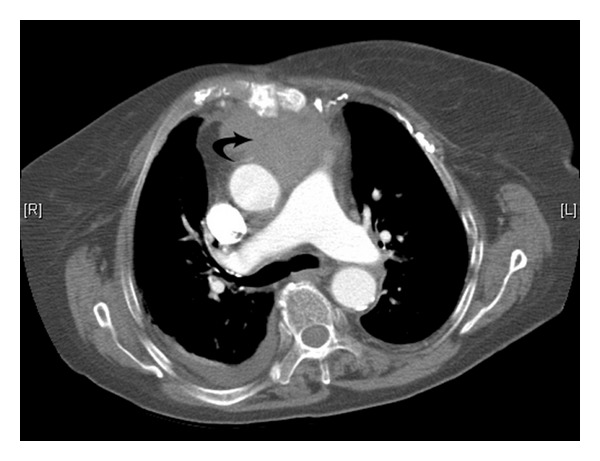
Axial slice from contrast-enhanced CT study depicting sternal osteomyelitis and a large related mediastinal collection (black curved arrow). The collection has higher density than that of water in keeping with pus and/or a degree of haemorrhage.

**Figure 2:**
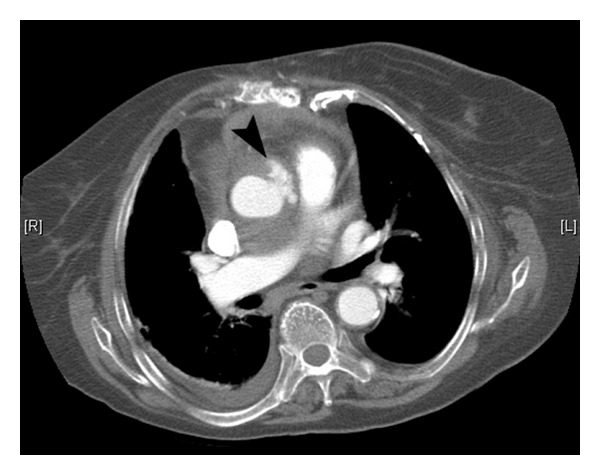
Axial slice from contrast-enhanced CT study demonstrating direct communication and erosion of the abscess cavity into the anterior ascending aorta resulting in a localised peri-aortic area of contrast extravasation (black arrowhead).

Initial treatment against the bacteraemia consisted of intravenous gentamicin and meropenem based on a working diagnosis of mediastinitis with or without bacterial endocarditis. The chest radiograph was unremarkable. Subsequent chest CT revealed osteomyelitis of the sternum, contiguous with a large anterior mediastinal abscess measuring 67 mm by 44 mm in diameter (Figure 1). The more interesting radiological feature was the direct communication and erosion of the abscess cavity into the anterior ascending aorta resulting in a localised periaortic area of contrast extravasation (Figure 2). The concomitant presence of endocarditis was ruled out on echocardiography which also revealed the large mediastinal cavity in close proximity to the aortic root as well as a 1.4 cm diameter pseudoaneurysm in the anterior ascending aorta.

Peridiagnosis, there were marked haemodynamic instability and a rapid decline. In view of her comorbidities, surgery was not deemed a suitable option and consequently the patient died soon after with pseudoaneurysm rupture as the attributed cause of death.

## 3. Discussion

Sternotomy wound complications following cardiothoracic surgery have been well documented in the medical literature and can have varied manifestations from sterile wound dehiscence to suppurative mediastinitis. Mediastinitis is often used synonymously to denote deep sternal wound infections, whereby wound infection is complicated by sternal osteomyelitis with or without the infective involvement of the retrosternal space. Wound discharge is the most common reported feature occurring in 70 to 90% of the cases, with other local manifestations including chest wall tenderness and sternal dehiscence.

Most cases of mediastinal wound infections are attributed to Gram-positive pathogens with *Staphylococcus aureus* and *Staphylococcus epidermidis* isolated in 70 to 80% of the cases [3] with *Pseudomonas* being the most reported Gram-negative causative organism. Gram-negative mediastinal infections though less frequently incriminated are often associated with a more complicated postoperative course. In addition these cases are often encountered with other concomitant “remote-site” infections most commonly pneumonias and urinary tract infections which are reported to cooccur in up to 58% of the cases postoperatively [6].

One such postoperative complication demonstrated by this case is the formation of a pseudoaneurysm. Pseudoaneurysms or false aneurysms involving the ascending aorta are rare sequelae of cardiac surgery with a reported incidence of less than 0.5% [4, 5]. These aneurysms represent a dreaded complication, with rupture as the most deleterious consequence. Postoperative development of aortic pseudoaneurysms is commonly encountered at sites of aortic wall disruption with aortotomy sites being the most commonly implicated following aortic valve replacement.

Several theories exist regarding the pathogenesis but infection is often heralded as the common denominator as in the case reported herein. Infected or mycotic pseudoaneurysms represent approximately 2.7% of all postsurgical aneurysms and are most often attributed to *Staphylococcus* and *Salmonella* species [7].

Early recognition and diagnosis of postoperative infective complications of cardiac surgery are imperative. Patients who have recently undergone open heart surgery that present with chest pain following any infection, even of a site and/or organism that is not usually associated with mediastinitis, should merit a high index of suspicion and should be investigated appropriately.

As demonstrated by this case, clinical improvement, negative blood cultures, and an unremarkable transesophageal echocardiography do not necessarily rule out an ongoing infective process and the development of complications such as a pseudoaneurysm.

## 4. Conclusion

Proteus mediastinitis is a rare complication of open cardiac surgery that can present late following initial clinical improvement and negative blood cultures and can result in potentially fatal pseudoaneurysm formation.

CT scanning has a role to play in the identification of these complications and should be considered prior to discharge in at-risk patients with sternal wound infections.

Postoperative vigilance for infective complications should be maintained after discharge and Gram-negative causative organisms such as *Proteus mirabillis* should be considered.

## References

[1] El Oakley RM, Wright JE (1996). Postoperative mediastinitis: classification and management. *Annals of Thoracic Surgery*.

[2] Gårdlund B, Bitkover CY, Vaage J (2002). Postoperative mediastinitis in cardiac surgery - Microbiology and pathogenesis. *European Journal of Cardio-Thoracic Surgery*.

[3] Demmy TL, Park SB, Liebler GA (1990). Recent experience with major sternal wound complications. *Annals of Thoracic Surgery*.

[4] Taylor PC, Groves LK, Loop FD, Effler DB (1976). Cannulation of the ascending aorta for cardiopulmonary bypass. Experience with 9,000 cases. *Journal of Thoracic and Cardiovascular Surgery*.

[5] Katsumata T, Moorjani N, Vaccari G, Westaby S (2000). Mediastinal false aneurysm after thoracic surgery. *Annals of Thoracic Surgery*.

[6] Rodríguez-Hernández MJ, De Alarcón A, Cisneros JM (1997). Suppurative mediastinitis after open-heart surgery: a comparison between cases caused by Gram-negative rods and by Gram-positive cocci. *Clinical Microbiology and Infection*.

[7] Malouf JF, Chandrasekaran K, Orszulak TA (2003). Mycotic aneurysms of the thoracic aorta: a diagnostic challenge. *American Journal of Medicine*.

